# Trends in Fall-Related Traumatic Brain Injury among Older Persons in Connecticut from 2000–2007

**DOI:** 10.4172/2167-7182.1000168

**Published:** 2014-07-31

**Authors:** Terrence E Murphy, Dorothy I Baker, Linda S Leo-Summers, Mary E Tinetti

**Affiliations:** Department of Internal Medicine, Section of Geriatrics, Yale University School of Medicine, New Haven, CT, USA

**Keywords:** Connecticut collaboration for fall prevention, Fall-related traumatic brain injury, Hospitalization, Emergency department, Anticoagulation therapy

## Abstract

**Background:**

Anecdotal evidence suggests a rising trend in the occurrence of fall-related traumatic brain injuries (FR-TBI) among persons ≥ 70 years. To document this apparent trend on a more substantive basis, this report longitudinally describes overall and age-stratified rates of three outcomes attributed to FR-TBI among persons ≥ 70 years: emergency department visits (ED), hospitalizations, and terminal hospitalizations.

**Methods:**

Eight years (2000–2007) of observational data from emergency departments and acute care hospitals serving a non-randomly selected, densely populated region in southern Connecticut, U.S.

**Results:**

From 2000–2007 among persons 70 years and older, overall rates of FR-TBI visits to emergency departments more than doubled while corresponding rates of hospitalization and terminal hospitalization rose 58% each. The point estimate of growth in the rate of ED in the oldest stratum was nearly triple that of the younger stratum whereas point estimates of growth in rates of hospitalization and terminal hospitalization were nearly four times higher. Total Medicare costs for ED visits increased nearly four-fold while corresponding costs for hospitalizations and terminal hospitalizations rose by 64% and 76%. The most common discharge diagnoses for ED and hospitalization were unspecified head injury and intracranial hemorrhage.

**Conclusions:**

The rapid rise in rates of FR-TBI and associated Medicare costs underscore the urgent need to prevent this burgeoning source of human suffering and health care utilization. We believe the rise in rates is at least partially due to a greater public awareness of the outcome that has been facilitated by increasing use of diagnostic imaging in the ED and hospital.

## Introduction

In the United States (U.S.) falls are the leading cause of traumatic brain injury (TBI) in persons aged 65 and older [1, 2]. U.S. national data between 2002 and 2006 showed that the rate of TBI-related hospitalization from unintentional falls among persons age 75 and older per 100, 000 population (339.3) was at least three times the rate of any other age group [3]. Several factors emphasize the need to better understand and prevent fall related traumatic brain injury (FR-TBI) among older adults. Foremost, these injuries result in long acute care hospitalization [4], longer periods of post-acute rehabilitation [5, 6], low likelihood of regaining pre-fracture functional ability [6, 7], and a high risk of recurrent fall-related injury [8]. For these and other reasons the need to study FR-TBI in older adults is urgent [2]. There have been, however, very few recent studies specifically reporting on FR-TBI [9–11].

Because of its mandated use of ICD-9-CM and E-codes to document injury-related hospital admissions and visits to the emergency department (ED), the state of Connecticut (U.S.) provides an opportunity to examine longitudinal trends in rates of FR-TBI among older persons. Combined with data from the corresponding Medicare population, this accounting of hospital data enables longitudinal analyses of three FR-TBI outcomes: visits to the emergency department not resulting in hospitalization (ED), admissions to hospital, and that subset of hospitalization admissions that terminated in either death or discharge to hospice care. This report describes overall and age-stratified rates of these three outcomes in southern Connecticut over the years 2000–2007, associated Medicare costs, and dominant ICD-9-CM codes. All rates and costs reported here are unadjusted, meaning they are not generated from statistical or econometric models, and therefore do not test for the significance of associations with covariates such as age or sex.

## Materials and Methods

### Study design

**Figure 1:**
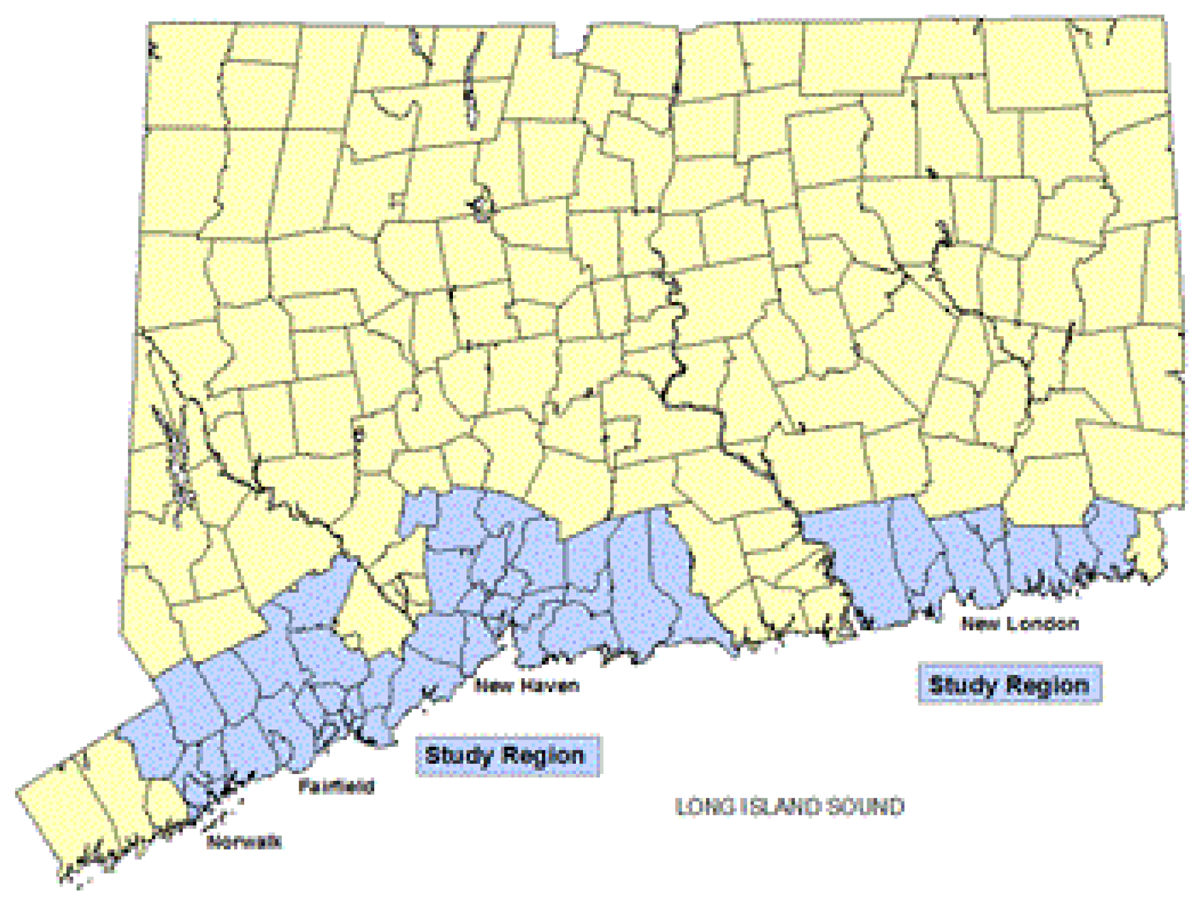
Study Region in Southern Connecticut for Evaluating Longitudinal Trends in Fall-Related Traumatic Brain Injury from 2000–2007

The Connecticut Collaboration for Fall Prevention (CCFP) is a statewide effort to move evidence regarding the prevention of falls into practice [12, 13]. The usual care area from the CCFP is the study region in this analysis, a discontinuous chain of 53 Zip Code Tabulation Areas along the Connecticut coastline (Figure 1). Seven acute care hospitals serve the study region, including one of the state’s two Level I Trauma Centers [14]. Because in the years 2000–2006 this area of the state was unexposed to CCFP’s fall-prevention programs, it depicts trends in these outcomes that are, for the most part, not influenced by any ongoing intervention. We did not include data more recent than 2007 for two reasons. The first is that at some time in 2007, a state sponsored program commenced with systematic dissemination of educational materials from CCFP in the study area [13]. The second reason is that the aggregation of regional data specifically focused on fall-related injury from different hospitals is complicated and costly.

### Outcome data

Outcome data regarding age, discharge diagnoses and hospital costs from the seven acute care hospitals in the study region were obtained from the Connecticut Health Information Management (CHIME) database, [15]. Our three outcomes are FR-TBI visits to the ED without hospitalization, admission to hospital, and that subset of hospital admissions terminating with death or discharge to hospice, i.e., terminal hospitalizations. We include those persons whose residential address corresponds to the Zip Code Tabulation Areas within the study region. We defined FR-TBI as any visit to the ED or hospital admission assigned a fall-related E-code by CHIME (817, 824, 880–888, or 927) and at least one of the following ICD-9-CM codes specific to TBI (800–801, 803–804, 850–854, or 959.01) [3].

### Population data

The at-risk population consisted of all persons 70 years and older residing in any of the 53 Zip Code Tabulation Areas comprising the study region. Estimates of the at-risk population for each year were derived from annual Medicare Denominator files.

### Statistical analysis

Age-standardized annual rates of visits to the ED, admissions to hospital, and terminal hospitalizations attributed to FR-TBI from all eligible persons were plotted across the eight year period. The numerators for each rate were the counts of events reported by CHIME while the denominators were the counts of persons 70 years and older living in the study area, as reported in the annual Medicare Denominator files. Age adjustment was by the direct standardization method using the 2000 Census population of the 53 Zip Code Tabulation Areas in five year age groups (70–74, 75–79, 80–84, 85 and over). Age-stratified rates (70–79 and ≥ 80 years) for each outcome were plotted. Descriptive statistics and associated Medicare costs (in 2007 U.S. dollars) were also tabulated on a yearly basis for each outcome. The frequencies of the dominant ICD-9-CM codes used to characterize visits to the ED and admissions to hospital over the eight years were also examined. Because we preferred to report trends that were not influenced by modeling assumptions, no model-based analysis of the reported rates and costs was undertaken.

## Results

The study region included over 109, 413 residents of age ≥ 70 years who were 61.4% female, 92.3% white, 5.8% black, 2.2% Hispanic, and with 63.8% reporting education of high school or less. It includes seven acute care hospitals, with a median capacity of 335 (range 65–897) staffed beds per hospital.

**Figure 2:**
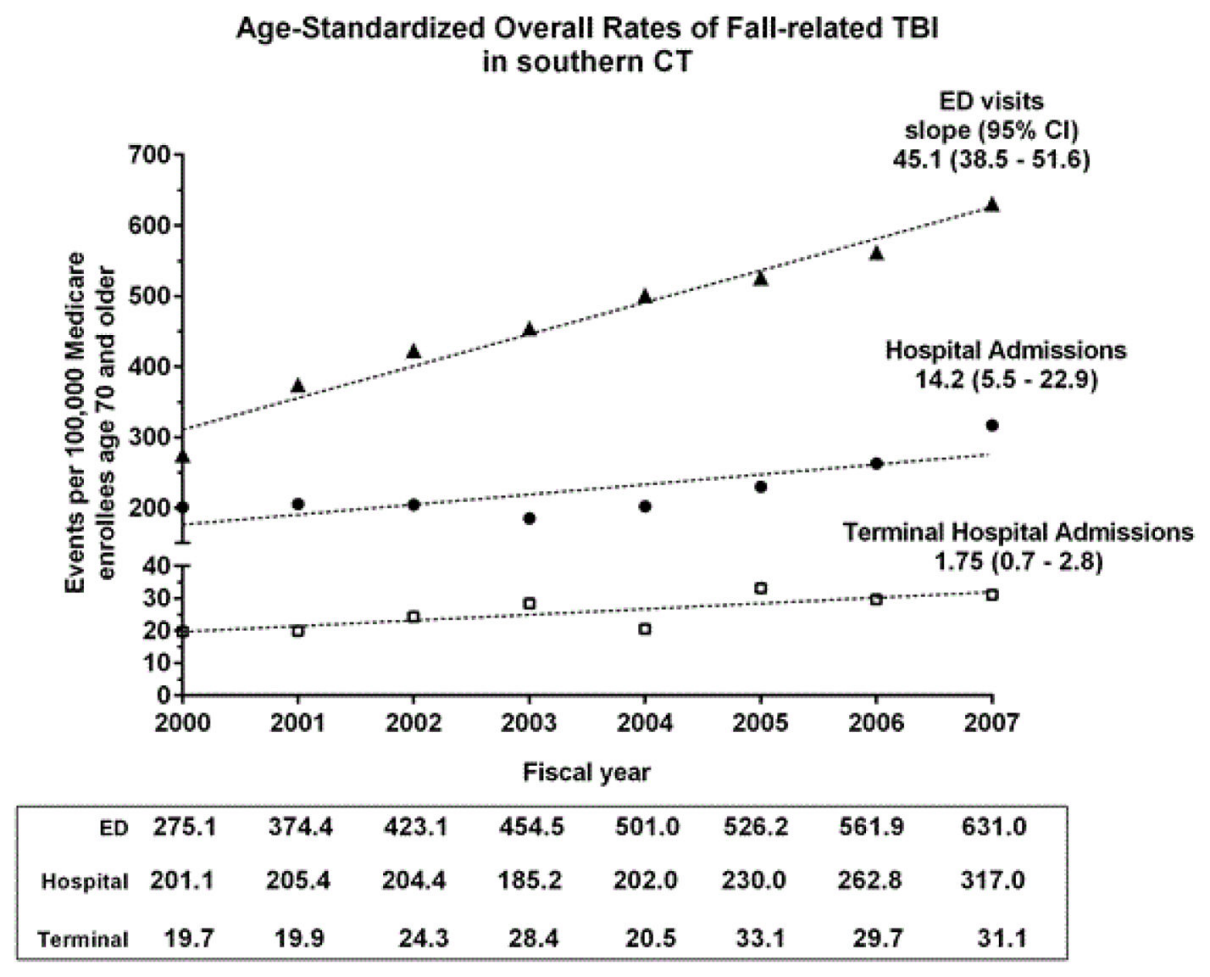
Overall Rates of Three Outcomes of Fall-Related Traumatic Brain Injury in Southern CT from 2000–2007

Figure 2 presents the age-standardized overall rates of visits to the ED, of admissions to hospital, and of terminal hospitalizations attributed to FR-TBI among persons 70 years and older residing in the study region. Between 2000 and 2007 the overall rate of FR-TBI visits to the ED for person’s ≥ 70 years of age more than doubled, while overall hospital admissions and terminal hospitalizations each rose approximately 58%. Of all FR-TBI hospitalizations, roughly ten percent terminated in death or discharge to hospice. We observe that because the 95% confidence intervals (CIs) of the slopes of each outcome are non-overlapping, the average growth in each outcome differs significantly from the others.

**Figure 3:**
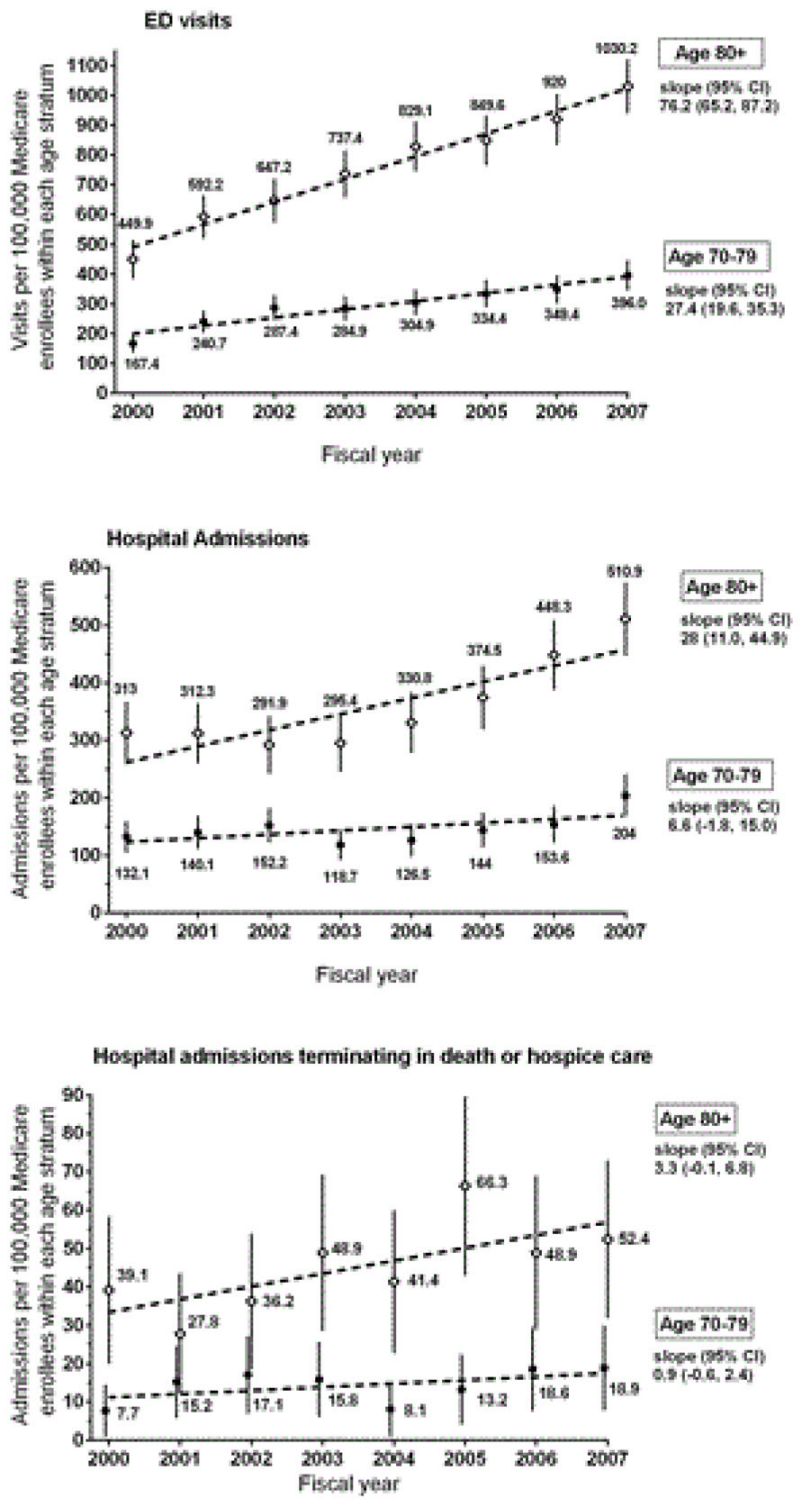
Age Stratified Rates of Three Outcomes of Fall-Related Traumatic Brain Injury in Southern Connecticut from 2000–2007

Figure 3 presents age-stratified (70–79 versus ≥ 80 years) annual rates for the three FR-TBI outcomes. We note the pronounced growth of these outcomes among the oldest old, defined here as ≥ 80 years of age. Because the CIs of the rates for ED visits and total hospital admissions are non-overlapping at each year, the rates of these two outcomes are consistently and significantly higher for the older stratum.

Table 1 indicates that median lengths of stay in days for both types of FR-TBI hospital admissions were nearly the same in 2007 as in 2000. It also shows that between 2000 and 2007 median cost for a FR-TBI visit to the ED rose by 75%, whereas median cost of FR-TBI hospital admissions rose only 1.6%. While pinpointing the causes of these proportional changes in cost is elusive, we speculate that the larger percent rise in ED costs is, to a certain degree, due to the growing availability of diagnostic imaging, such as CT scans and MRI, in the ED. Relative to hospital admissions, the larger rise in ED cost is also in part attributable to the relative sizes of the median ED and hospital costs at baseline. In 2000 the median cost of ED in 2000 was $650, compared to $9, 150 for hospital admission. This means that whereas an increase of several hundred dollars represents a large proportional change in the cost of ED, it represents little change in the overall cost of a hospital admission.

We ascribe the large, longitudinal variability in median cost of terminal hospitalization to several factors. First, each yearly observation is based on a small number of events, which implies larger sample variability. Second, those TBIs not immediately resulting in death often require long, multi-disciplinary interventions where patients are moved from life support to less acute care. Because this necessarily involves consultation with teams of specialists and the patient’s family, the process of treating a terminal patient is complicated. These details add cost and variability to the patient’s treatment for FR-TBI.

Table 1 also shows that total costs for FR-TBI visits to the ED rose nearly four-fold over this same period whereas total costs for all hospital admissions and terminal hospitalizations rose by 64% and 76%. The respective rises in overall costs largely reflect the growth in frequency of each type of event over time.

Finally, the most frequent ICD-9-CM code reported for ED visits over the eight years was code 959.01 (unspecified head injury), accounting for 87.3% of all FR-TBI discharge diagnoses. The next two codes were 850 (concussion) and 852 (subarachnoid, subdural, and extradural hemorrhage, following injury), respectively accounting for 7.9%, and 1.6% of ED discharge diagnoses. For hospital admissions, code 852 (subarachnoid, subdural, and extradural hemorrhage, following injury) accounted for 45.5% of FR-TBI discharge diagnoses. The next most frequent codes were 851 (cerebral laceration and contusion), 959.01 (unspecified head injury), 850 (concussion), 801 (fracture of base of skull), and 853 (other and unspecified intracranial hemorrhage following injury). These codes respectively accounted for the following percentages of FR-TBI hospital discharge diagnoses: 13.2%, 11.5%, 9.2%, 8.5%, and 8.1%.

Of the 235 hospitalizations attributed to FR-TBI that terminated in death or discharge to hospice, the most frequent ICD9-CM codes were the following: 852 (subarachnoid, subdural, extradural hemorrhage following injury), 851 (cerebral laceration and contusion), 853 (other and unspecified intracranial hemorrhage following injury), 801 (fracture of base of skull), 850 (concussion), 800 (fracture of vault of skull), and 803 (other skull fracture). They accounted for the following percentages of all FR-TBI terminal hospitalization discharge diagnoses: 55.7%, 12.6%, 11.7%, 8.0%, 3.4%, 2.9%, 2.5%, and jointly represent 96.7%.

## Discussion

The major findings of this study are the rapidly increasing rates and costs of emergency and hospital care for older adults who have sustained FR-TBI. Hospital admissions were largely attributed to a small number of ICD9-CM codes, primarily code 852 (subarachnoid, subdural, and extradural hemorrhage, following injury). Previous research provides mixed results regarding any suggested association between intracranial hemorrhage and treatment with anticoagulants, with some suggesting higher risk of intracranial hemorrhage in patients 80+ years of age [16] and others suggesting a protective association against a composite outcome comprised of stroke, intracranial hemorrhage, myocardial infarction, and death [17]. Given the uncertainty regarding the association between anticoagulation and risk of FR-TBI, its use in the very old population must include close monitoring.

While it would be prohibitively difficult to rigorously identify the reason for the rising rate of FR-TBI among older persons in this part of Connecticut, we believe it is somewhat explained by growing public awareness. Because the population of persons 80 years and older is growing rapidly, there is a larger number of events being documented, which subsequently reminds clinicians to think more frequently about this outcome. Due to growing access to diagnostic imaging in the ED, the ability to objectively detect these head injuries has been boosted. There is likewise a growing awareness that because older persons are innately fragile, the same fall that causes an injury such as hip fracture may have also concurrently produced a TBI.

On a related note, while there is some evidence that community-based fall prevention efforts can reduce the rate of FR-TBI hospital admissions, [18] a better understanding of the circumstances preceding these admissions to hospital, with particular attention to those over 80 years of age, could help refine assessment and focus prevention efforts [19].

Our study has several limitations that should be noted. This was an observational study of a study region originally designed to match the treatment region of the Connecticut Collaboration for Fall Prevention, therefore not a random sample of the state. Because we did not have access to patient specific information, our estimated rates of outcomes assume that each event is distinct, even though some may be repeat visits to the ED or hospital for the same event. We cannot determine the extent to which hospitalizations terminating in death were attributable to FR-TBI versus other comorbidities. For example, among persons receiving hospice care, falls are a common reason for visits to the ED and admission to hospital. Because the hospice patients are already terminally ill, any of these patients who died from a FR-TBI that took place in hospice confounded our estimated rates of terminal hospitalization [20].

Despite these limitations, the study provides a detailed, longitudinal analysis of three important FR-TBI outcomes among older persons. It also corroborates the rising rate of FR-TBI among older persons observed in regional studies in Canada, [9] Australia, [11] and the Netherlands [10].

## Conclusions

The rapid growth in Medicare costs for FR-TBI warrants concern. In 2000 direct medical costs of fatal and non-fatal falls among older adults from ED visits and hospital admissions in the U.S. were estimated to total $0.2 billion and $19 billion, respectively [21]. If the overall costs of fall related injury have grown like those documented here, this surging financial burden is a national concern. Although we partially attribute these trends to growing public awareness, the rapid rise in rates of FR-TBI and associated Medicare costs among older persons in this portion of Connecticut underscores the urgent need to understand and prevent this burgeoning source of human suffering and health care utilization.

## Figures and Tables

**Table 1:** Characteristics of Fall Related Traumatic Brain Injury Outcomes in Southern Connecticut (2000–2007): ED Visits, Hospital Admissions, and that Subset of Hospital Admissions Terminating in Death or Hospice Care

Characteristics	Medical Service	FY00	FY01	FY02	FY03	FY04	FY05	FY06	FY07
Length of Stay	Hospital admission	6	5	5	5	5	5	4	5
Median (Q1, Q3)		(4, 12)	(3, 10)	(3, 9)	(3, 10)	(3, 8)	(2, 8)	(3, 9)	(3, 9)
Days	Terminal Hospitalization^b^	3	4	6	5	5	4	7	4
		(2, 11)	(1, 9)	(1, 15)	(2, 12)	(2, 11)	(2, 11)	(2, 15)	(2, 14)
Median Cost (Q1, Q3) per Stay (thousands of dollars)^a^	ED	0.65	0.7	0.76	0.82	0.91	0.98	1.06	1.14
		(0.35, 1.0)	(0.4, .99)	(0.46, 1.0)	(0.59, 1.1)	(0.67, 1.3)	(0.75, 1.3)	(0.74, 1.4)	(0.83, 1.5)
	Hospital admission	9.15	8.32	8.22	8.8	8.03	8.29	8.65	9.3
		(5.3, 16.8)	(4.5, 15.4)	(5.0, 14.6)	(5.2, 17.0)	(4.8, 14.4)	(4.9, 16.3)	(5.4, 16.1)	(5.6, 17.4)
	Terminal Hospitalization^b^	4.83	7.45	10.92	13.11	8.17	7.74	16.22	13.37
		(3.3, 22.2)	(5.2, 15.4)	(5.2, 26.9)	(5.0, 26.9)	(5.4, 18.1)	(4.9, 24.3)	(7.0, 39.2)	(7.8, 36.9)
Total Cost for All Stays (millions of dollars)^a^	ED	0.22	0.32	0.38	0.46	0.58	0.65	0.71	0.87
	Hospital admission	3.49	3.31	3.07	3.04	2.9	3.81	4.1	5.71
	Terminal Hospitalization^b^	0.5	0.33	0.59	0.58	0.42	0.72	0.76	0.88
Number of Events	ED	293	414	471	512	569	599	640	723
	Hospital admission	214	227	227	208	230	262	302	363
	Terminal hospitalization	21	22	27	32	24	39	34	36

a 2007 U.S. Dollars,

b death while hospitalized or discharge to hospice, Q1=1^st^ quartile, Q3=3^rd^ quartile, ED=visit to the Emergency Department, FR-TBI=Fall-Related Traumatic Brain Injury, FY00=fiscal year 2000, October 1999 through September 2000
